# Flexible and Wearable Biosensors for Monitoring Health Conditions

**DOI:** 10.3390/bios13060630

**Published:** 2023-06-07

**Authors:** Zhimin Song, Shu Zhou, Yanxia Qin, Xiangjiao Xia, Yanping Sun, Guanghong Han, Tong Shu, Liang Hu, Qiang Zhang

**Affiliations:** 1 Department of Anesthesiology, The Second Hospital of Jilin University, Changchun 130041, China; 2 Department of Anesthesiology, Jilin Cancer Hospital, Changchun 130021, China; 3 State Key Laboratory of Electroanalytical Chemistry, Changchun Institute of Applied Chemistry, Chinese Academy of Sciences, Changchun 130022, China; 4 School of Biomedical Engineering, Guangdong Laboratory of Artificial Intelligence and Digital Economy (SZ), Shenzhen Key Laboratory for Nano-Biosensing Technology, International Health Science Innovation Center, Research Center for Biosensor and Nanotheranostic, Health Science Center, Shenzhen University, Shenzhen 518060, China; 5 Department of Oral Geriatrics, Hospital of Stomatology, Jilin University, Changchun 130021, China; 6 State Key Laboratory of Radiation Medicine and Protection, School for Radiological and Interdisciplinary Sciences (RAD-X) and Collaborative Innovation Center of Radiation Medicine of Jiangsu Higher Education Institutions, Soochow University, Suzhou 215123, China

**Keywords:** wearable sensors, E-skins, sweat sensors, self-powered biosensors

## Abstract

Flexible and wearable biosensors have received tremendous attention over the past decade owing to their great potential applications in the field of health and medicine. Wearable biosensors serve as an ideal platform for real-time and continuous health monitoring, which exhibit unique properties such as self-powered, lightweight, low cost, high flexibility, detection convenience, and great conformability. This review introduces the recent research progress in wearable biosensors. First of all, the biological fluids often detected by wearable biosensors are proposed. Then, the existing micro-nanofabrication technologies and basic characteristics of wearable biosensors are summarized. Then, their application manners and information processing are also highlighted in the paper. Massive cutting-edge research examples are introduced such as wearable physiological pressure sensors, wearable sweat sensors, and wearable self-powered biosensors. As a significant content, the detection mechanism of these sensors was detailed with examples to help readers understand this area. Finally, the current challenges and future perspectives are proposed to push this research area forward and expand practical applications in the future.

## 1. Introduction

Personal medicine, also named precise medicine, treats patients based on various personal characteristics, which will be the next developing direction of health science and modern medicine. Earlier diagnosis and timely monitoring are incredibly important methods to reduce patients’ suffering and mortality. Although centralized healthcare service including hospitals is the primary choice for disease diagnosis, it is inconvenient and time/cost-consuming for most people [1]. In recent years, flexible and wearable devices were developed to monitor health status in real time, which provides a method to compensate for some shortcomings of centralized healthcare services [1,2]. Wearable biosensors can convert physiological signals to measurable electrical signals in real time such as current, capacitance, and resistance that reflect specific health information [3,4]. Compared with expensive and bulky equipment, wearable biosensors exhibited the characteristics of convenient applications, timely display, portability, and low cost. Wearable biosensors have found applications in monitoring pulse beat [5,6], metabolites in body fluids [7,8], temperature changes [9], cardio activities [10,11], etc. For example, a mechanically adaptable capacitive hydrogel-based sensor was fabricated by Wu and co-workers using a bioinspired mineral ionic hydrogel, which shows the characteristic of high sensitivity (0.17 kPa^−1^), and it shows potential applications in the real-time detection of human motions, such as finger touch and pulse beat [12]. In addition, multifunctional biosensors are desirable to achieve multiple applications. In one case, Cho and co-workers reported a bimodal wearable electronic device, which can achieve temperature/pressure detections in real time. The sensor was fabricated by utilizing reduced graphene oxide (rGO) as a thermistor in the capacitive pressure sensor, which has shown a sensitivity of 0.7 kPa^−1^ and 0.83% K^−1^ for pressure and temperature, respectively [9].

Although a lot of progress has been achieved in recent years, the research on wearable biosensors is still at the nascent stage. Sensitivity is still a general challenge for practical applications due to small changes in physiological pressure or metabolite concentration in body fluids. For example, the ejection of blood from the ventricle into arteries caused vasodilation behaviors and small pressure changes, which reflects cardiovascular health information [2,13]. The concentration of most metabolites such as uric acid, lactate, and ions in sweat is as low as μM levels [1,12]. Thus, the detection of these low physiological signals and metabolites requires sensors with high sensitivity. A series of methods have been employed to increase the sensitivity of wearable sensors such as utilizing nanomaterials [14,15], using enzymatic reactions [16,17], fabricating micropatterns [5,18,19], etc. For example, Park and co-workers employed a hybrid nanofibrous membrane of Ti_3_C_2_Tx MXene and poly(vinyl alcohol) elastomer to fabricate wearable biosensors [20]. The effective contact surface and dielectric property of the capacitive pressure biosensors were both enhanced owing to the utilization of ionic nanomaterials. The sensor exhibited sensitivities of 5.5 kPa^−1^ and 1.5 kPa^−1^ in the ranges of 0–30 kPa and 30–250 kPa, respectively. In addition, fabricating micropatterns is another important method to increase the sensitivity of wearable biosensors. Meanwhile, various patterns with different sensing functions can be integrated into one device. For example, Shen and co-workers prepared an elastic micropattern array-based multiple functions sensor, which is capable of sensing pressure and temperature [21]. The micropattern arrays were prepared with single-walled carbon nanotubes/thermoplastic polyurethane film. The interlocked micropatterned arrays can concentrate external pressure on the narrow arrays, which improved the sensing performance. The wearable sensors exhibited a dual-sensing ability to detect pressure and temperature, which show a high sensing sensitivity of 0.02 kPa^−1^ and 1.65% C^−1^ for pressure and temperature, respectively.

The outstanding capabilities of wearable biosensors have been illuminated in several recent reviews including non-invasive [22,23], multiple metabolites analysis [24,25], on-line monitoring [26,27], etc. However, this paper covers the aspects of detection objects, preparation methods, application manners, signal processing, and practical applications. It differs from others with the contents of advanced micro-nanofabrication technologies, piezoelectric sensors, and self-powered sensors that are rarely mentioned in previous reviews. Firstly, the common bio-fluids that can provide target analytes in wearable sensors and various types of structures suitable for human wearing are pointed out. Then, the main preparation methods of wearable sensors are introduced to explain the relationship between sensor structures and sensing functions. Then, various wearable biosensors based on their applications will be discussed including wearable physiological pressure sensors, wearable sweat sensors, and wearable self-powered biosensors. In addition, the sensing mechanism will be introduced along with these recently published examples. Finally, future perspectives and challenges in this field will be proposed to guide future research in this area. We hope this review can familiarize readers with the most cutting-edge achievements and encourage more researchers to devote more efforts to push forward the development of wearable biosensors.

## 2. Detection Objects

### 2.1. Saliva

Saliva is secreted by the parotid glands composed of mucosal exudates, gastric reflux, and gingival crevicular fluid. Massive important biomarkers are found in saliva including drug metabolites, microorganisms, proteins, hormones, etc. [28]. Due to the exchange between salivary glands and blood, saliva analysis can be used to substitute blood analysis due to the merits of convenient collection and non-invasive manner [29]. A series of biosensors have been demonstrated to monitor pH, glucose, electrolytes, sulfuret, salivarius, etc. [30,31,32,33,34]. In addition, saliva analysis is well-known for COVID-19 detection [35].

### 2.2. Sweat

Sweat is an important biological fluid secreted by sweat glands distributed throughout the whole body. The sweat composition is related to blood with a much lower concentration. It has been reported that sweat contains abundant biomarkers such as glucose [36], cortisol [37], electrolytes [38], uric acid [39], etc. As such, sweat is one of the most widely studied analysis objects for wearable sensors [40,41,42].

### 2.3. Tears

Tears are composed of water, proteins, electrolytes, sugars, organic acids, etc. [43]. Variations in tear components can reflect the health condition of the eyes, which is incredibly important for the diagnosis and treatment of eye diseases [44]. For example, the reduction in mucin or lactoferrin concentration in tears has been used to diagnose dry eyes [45].

### 2.4. Interstitial Fluids (ISFs)

ISF results from the permeation of blood plasma from capillaries, which serves as a complementary biological fluid. The capillaries can dynamically exchange signaling molecules and metabolites with surrounding tissues, which leads to various biomarkers in ISF [46]. Since the ISF is located under the skin, a microneedle structure is generally required to conduct sample collection and complete detection [47,48,49]. Here, a wearable biosensor was fabricated to detect β-hydroxybutyrate (HB) and glucose in ISF for monitoring diabetic ketoacidosis conditions. β-hydroxybutyrate dehydrogenase and glucose oxidase were immobilized on the surface of microneedles, which can sense HB and glucose through an enzymatic reaction by piercing skins [50].

### 2.5. Respiration

It has been reported that exhalation contains as many as 870 kinds of volatile organic compounds (VOCs) [51]. These VOCs are produced by various biochemical processes, which are capable of providing important information about metabolic disorders or dysfunction [52]. Methanol and chloroform are recognized as the disease markers usually released due to the central nervous system, liver, lungs, and kidneys. A chemical sensor was prepared with graphene and metal–organic frameworks, which have a high sensitivity and selectivity for sensing chloroform and methanol [53].

### 2.6. Physiological Pressure

Physiological pressure is another important physiological signal resulting from activities, which can reflect the health levels of the human body. For example, blood pressure is an important method to evaluate cardiovascular diseases [54]. Intraocular pressure is a key index to monitor the risk and condition of glaucoma patients [55]. Wearable sensors have been used as a non-invasive way to monitor physiological pressures [56]. Various sensing mechanisms have been utilized to convert physiological pressures into bioelectric signals such as capacitive, resistive, triboelectric, and piezoelectric to realize the purpose of health monitoring [57]. Physiological pressure spans a very large range dependent on body parts and specific activities. In general, intraocular and intracranial pressures are less than 10 Pa, while blood pressure and vocal cord vibration belong to the medium pressure range (10–10 kPa) [58]. Therefore, the sensing pressure range is an important factor for wearable pressure sensors to use in the real world. High sensitivity in a wide range is another important factor to evaluate the performance of wearable pressure sensors. Much effort has been devoted to obtaining high sensitivity in a broad range [59,60]. For example, a flexible pressure sensor was constructed with fiber microspheres [59]. The fiber structure enables the sensor to have the capability of withstanding high pressure, which yields a high sensitivity in an ultra-wide linear range. The sensor has been used to detect various physiological signals including pulse, vocalization, and joint torque in a full range.

The detection of biomarkers in various biological fluids with wearable sensors has attracted extensive attention from researchers [61,62,63,64]. Up to now, blood is still the most important biological fluid for health diagnosis, but research attention starts to shift to non-invasive fluids, such as sweat, tears, saliva, and ISF. These biological fluids have similar components to blood, which can be acquired in a non-invasive manner, thus avoiding the risk of infection. However, the various relationships between biomarkers in these biological fluids and health conditions are still not clear, which limits the clinical and commercial applications of wearable sensors. The analysis of biomarkers by gold standards is essential to obtaining reliable clinical disease diagnosis standards, which can further advance the development of wearable sensors.

## 3. Preparation Methods

### 3.1. Surface Processing

**Figure 1:**
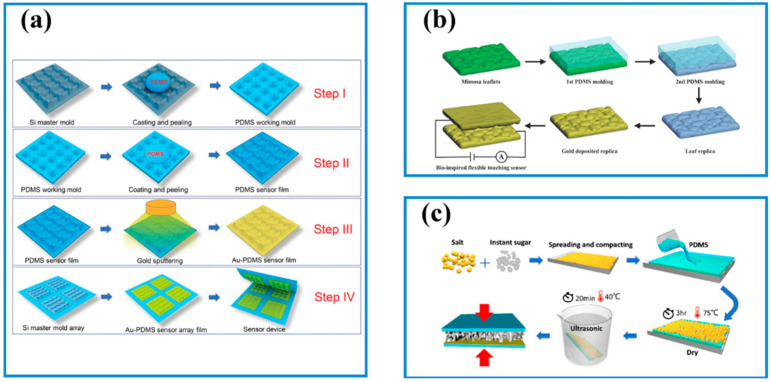
Schematic illustration of typical surface processing. (**a**) PDMS molds. Reproduced with permission from [75]. Copyright 2020, The Authors. (**b**) Natural plants. Reproduced with permission from [76]. Copyright 2014, WILEY-VCH Verlag GmbH & Co. KGaA, Weinheim. (**c**) Salt and instant sugar as templates. Reproduced with permission from [77]. Copyright 2019, American Chemical Society.

Surface processing provides an effective way of modifying bulk materials and electrodes to improve sensing performance such as sensitivity, limit of detection (LOD), selectivity, etc. Many attempts have been made to obtain a functional bulk matrix for sensing and biosensing, such as dip coating, drop casting [65], spray coating [66], electrochemical deposition [67,68], and printing [69]. Dip coating is a general strategy with the assistance of surface pre-treatment to obtain functional surfaces. A series of conductive coatings have been deposited onto commercial porous insulated substrates (e.g., foam [70], sponge [71,72], textile [73]) by dip coating, which provides a route to prepare electronic sensors. Template methods are an important method that can be used to prepare patterns on both rigid and flexible substrates [74]. For example, a flexible rectangular pyramids Au electrode on polydimethylsiloxane (PDMS) film was prepared using a commercial silicon microneedle array, as shown in Figure 1a. Firstly, the mixture of PDMS precursors was cast on commercial silicon microneedle arrays. After completely being cured, the PDMS film with concave microstructures was peeled off and used as a template. Then, uncured PDMS was cast on the template and cured to obtain the rectangular pyramid structure. Finally, an Au nanofilm was deposited on the film to fabricate conductive electrodes with the rectangular pyramid structure [75]. In addition, some plant leaves have unique microstructures (e.g., lotus leaves, natural mimosa leaves), which have been used as templates to form these microstructures on electrodes (Figure 1b) [76]. Another method has been reported to create a random microstructure using some soluble powers such as sugar and salt powders and so on [77,78,79]. A pre-spinning PDMS solution was mixed with salt granules and agglomerated instant sugar powder. Once the salt granules and sugar particles were dissolved, a micropattern formed throughout the PDMS layer (Figure 1c) [77]. Although the controllability of the patterning microstructure is imprecise, this work provides a quite easy, efficient, and low-cost method.

In addition to physical processing, chemical modification is robust, which can be well designed according to the analytes of interest. Some functional groups can be immobilized on the surfaces of sensing materials via chemical processing, such as hydroxyl, carbonyl, carboxylic acid, and so on [80,81,82]. These functional groups were used as reactive sites to bind with a variety of receptors that can selectively capture the analytes for sensing. For example, Neethirajan and co-workers prepared an rGO embedded wearable screen-printed electrode for the detection of cortisol and lactate in human fluids such as sweat and saliva [82]. The fabrication process is composed of the following two steps: firstly, the rGO was deposited on screen-printed electrodes by chemical processing. Then, the cortisol and lactate antibodies were bioconjugated to rGO on the surface of the screen-printed electrodes using covalent carbodiimide chemistry. The sensor can quantitatively detect cortisol and lactate using the electrochemical chronoamperometric method, which exhibited the detection limitations of 0.1 ng mL^−1^ and 0.1 mM for cortisol and lactate, respectively. Meanwhile, this method also improved the surface wettability and changed the surficial morphology, both of which increased the sites for binding analytes and thus improved the sensing performance.

### 3.2. Micromanufacturing Technologies

**Figure 2:**
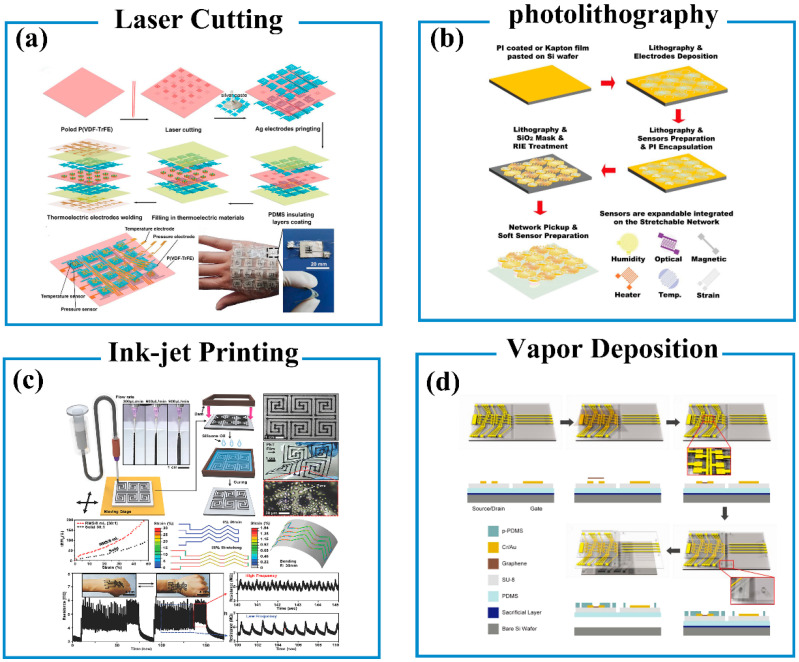
(**a**) Fabrication process flow of the active bimodal sensor array by laser cutting. Reproduced with permission [83]. Copyright 2020, Wiley-VCH GmbH. (**b**) Fabrication process of stretchable and conformable matrix network by photolithography. Reproduced with permission [86]. Copyright 2018, The Author(s). (**c**) Schematic diagram of the ink-jet printing process for the patterned PPSR sensor. Reproduced with permission. Copyright 2014, WILEY-VCH Verlag GmbH & Co. KGaA, Weinheim [89]. (**d**) Schematic representation of vapor deposition employed to fabricate pressure sensor arrays. Reproduced with permission [90]. Copyright 2017, The Author(s).

Several micromanufacturing technologies have been proposed to fabricate flexible and wearable biosensors, such as laser-cutting technology [83,84,85], photolithography technology [8,86], ink-jet printing technology [87,88,89], and vapor deposition [90,91,92,93]. Laser-cutting technology is a highly accurate material-processing technology, which uses high-density energy generated by laser focusing to etch materials for desirable patterns [94,95]. With laser-cutting technology, multifunctional biosensors can be prepared by integrating different sensor components into one device. For example, Deng and coworkers prepared a pressure/temperature bimodal tactile sensor array employing all organic functional materials by laser-cutting technology and screen printing (Figure 2a) [83]. The sensor array is composed of a pressure sensor and a temperature sensor. Laser cutting was utilized to rapidly and precisely punch the poly(vinylidene fluoride-co-trifluoroethylene) (p(VDF-co-TrFE)) piezoelectric film, which makes the p(VDF-co-TrFE) piezoelectric film with space hole pattern for further integrating thermoelectric pillars for sensing temperature. The temperature-sensing element is mainly made from five P–N couples, where the P leg is a polyaniline-based composite pillar and the N leg is an Ag paste pillar. Screen printing was utilized to connect Ag electrodes to the sensor array. The sensor array can convert pressure and temperature stimuli into two independent electric signals without interference based on the thermoelectric effect of polyaniline and the piezoelectric effect of p(VDF-co-TrFE), respectively. The sensor shows a high temperature-sensing sensitivity of 109.4 µV K^−1^ and a pressure-sensing sensitivity of 640 mV kPa^−1^, respectively.

Photolithography technology uses the principle of optic-chemical reaction to imprint patterns on the surface of a medium and achieves desired patterns with specific functions. It has been widely used to produce various patterns and microstructures with the characteristics of ease manipulation, high resolution, and precision [96]. It enables fabricating multifunctional biosensors by integrating various sensor units on a plate. For example, Wang and co-workers reported a skin-inspired highly stretchable, and conformable human somatosensory system by photolithography technology (Figure 2b) [86]. The specific sensor patterns and elements are sputtered on the substrate by photolithography to achieve multifunctional sensing networks, which can sense temperature, in-plane strain, humidity, light, magnetic field, pressure, and proximity. Javey and co-workers prepared a wearable microfluidic tactile diaphragm pressure sensor for real-time health monitoring, which is composed of embedded Galinstan microchannels and PDMS substrate [97]. The Galinstan-based equivalent Wheatstone bridge circuit was embedded in the PDMS substrate using photolithography technology, which improved the sensing performance. The sensor exhibited a sensitivity of 0.0835 kPa^−1^, low detection limits of <100 Pa, and a response time of 90 ms. It exhibited a potential application in health monitoring.

Ink-jet printing can be used to achieve a circuit design image on wearable devices by propelling ink droplets on flexible substrates, which minimizes materials waste and simplifies the processing procedure [98,99]. Kim and co-workers utilized an all-solution-based ink-jet printing technology to fabricate a wearable rubber pressure sensor by introducing pores into conventional pressure-sensitive rubbers (PSRs) (Figure 2c) [89]. The porous structure can increase the sensitivity of the sensor, which allows the sensor applications as human–machine interfaces for wirelessly controlling machines. The ink-jet printing technology also has been used to prepare flexible electrodes with the characteristics of low-cost, large-scale manufacturing, and good repeatability. A highly integrated hybrid stretchable electronic was fabricated by ink-jet printing with the two main features of double-side strain isolation and double-side electrical conduction [100]. The stretchable electrodes and rigid islands pattern were ink-printed on a PDMS soft substrate, which minimized the strain imposed on the chips.

Vapor deposition can be used to deposit thin films with a thickness of several to hundreds of nanometers that have been widely used in preparing wearable sensors and bioelectrodes for health monitoring [90,91,92,93]. Interdigital electrodes can be prepared by vapor deposition, which was used to fabricate field-effect transistors [90]. The air between the interdigital electrode and gate electrodes was used as a dielectric layer. When the pressure was applied to the gate electrode, the distance between the interdigital electrode and gate electrodes will decrease. The sensor exhibited a wide pressure sensing range (250 Pa to ~3 MPa) and high sensitivity (2.05 × 10^−4^ kPa^−1^), as shown in Figure 2d [90]. In another case, a high-performance MoS_2_ field-effect transistor on paper was fabricated using vapor deposition technology, which was used as a flexible and environmentally wearable electronic sensor [92]. The MoS_2_ pattern was deposited on sapphire substrates, which were then transferred to polystyrene substrates. In addition, vapor deposition is also widely utilized to pattern the electrodes for achieving the circuit of integrated flexible sensor arrays. For example, Gao and co-workers prepared fully integrated wearable sensor arrays by patterning the Au-based flexible electrodes on the skin temperature substrate using vapor deposition [8]. Thus, the sensor array exhibited the high-performance of electrochemically detecting the sweat metabolites (such as glucose and lactate), electrolytes (such as sodium and potassium ions), and skin temperature.

The advance in manufacturing technologies pushed the development of wearable sensors forward in the last decade. However, there are still some limitations in the view of both the technical side and theory research. The resolution on the nanoscale is still hard to manipulate and massively produce during sensor fabrication. The relationship between sensor performance and surface morphologies should be further investigated to provide more concepts for designing new sensors in the future. The development of new manufacturing technologies is indispensable to solve the aforementioned issues.

## 4. Application Manners

Wearable sensors have been designed into various structures and shapes to fit their practical applications. The typical shapes and patterns of wearable sensors will be discussed in this section.

### 4.1. Patch

**Figure 3:**
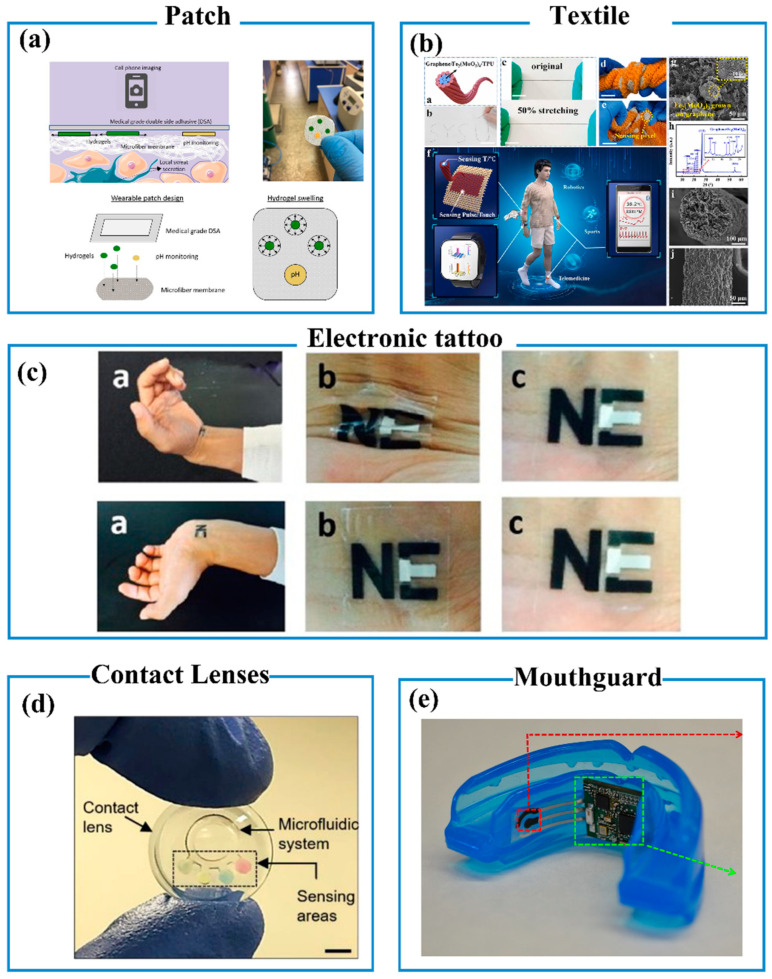
Application manners of wearable sensors. (**a**) Patch [101]. Copyright 2020, Elsevier B.V. (**b**) Textile. Copyright 2022, Elsevier Ltd. [105]. (**c**) Electronic tattoo [109]. Copyright 2014, Elsevier B.V. (**d**) Contact lenses [116]. Copyright 2020, Elsevier B.V. (**e**) Mouthguard [121]. Copyright 2015, Elsevier B.V.

The patch is one of the ideal choices for wearable sensors due to the feature of bendability and fitness to the skin surface. A bunch of patch-type wearable sensors was fabricated to realize various functions for health monitoring [101,102,103]. A skin patch was prepared using a microfiber membrane with sodium polyacrylate microgels to measure sweat rate and pH [101]. The sweat rate can be calculated from the changes in microgel volume. Several dyes have been incorporated into the microgels that show the colorimetric response for sweat pH (Figure 3a). Physiological signals are important information that is closely associated with health conditions. A compact wearable patch was integrated with a centerboard for signal processing and three sensors for vital signs monitoring [104]. It shows the capability of recording electrocardiogram (ECG), photoplethysmography, and body temperature.

### 4.2. Textile

Compared with planar wearable sensors, textile-based sensors exhibit the advantages of excellent air permeability, comfort, and perspirability. Cotton and polyester are commonly used in textile-based sensors. Since textile-based fibers are non-conductive, functional components should be weaved into textile-based fibers to sense health biomarkers. For example, a core–shell structured yarn was prepared by wrapping graphene/Fe_2_(MoO_4_)_3_ fibers with nylon monofilament, which was weaved with polyester textiles [105]. The thermoresistive graphene/Fe_2_(MoO_4_)_3_ fibers show the capability of detecting body temperature. Moreover, the whole textile can be used as a triboelectric nanogenerator to detect touch/pulse (pressure) in real time, as shown in Figure 3b [105]. In another case, cotton thread was modified with poly(3,4-ethylenedioxythiophene)-poly(styrenesulfonate (PEDOT:PSS) to produce a conductive performance, which was seamlessly integrated with textiles or clothing for monitoring heart activity [106]. No sensing performance loss was observed after 15 times of machine washability.

### 4.3. Wearable Tattoo Sensors

The electronic tattoo (e-tattoo) is an emerging category of wearable ultra-thin sensors with a thickness of <500 nm and optical transparency of >85% [107,108]. The e-tattoo can be printed on any body part of the wearers. The adhesive-containing device can be applied anywhere on the wearer’s skin adhered mainly by van der Waals forces. The ultra-thin thickness leads to breathable and stiffness-negligible features. The e-tattoo has shown the capability of monitoring a series of physiological information such as metabolites, body temperature, ECG, and electromyography [109,110]. For example, a tattoo-based sensor was printed on the arm of volunteers using a bismuth/Nafion film electrode, as show in Figure 3c [109]. It can monitor zinc in sweat through voltammetry during exercise with the resistance of repeated mechanical deformation.

### 4.4. Microneedles-Based Sensors

Microneedles-based sensors are another important type of wearable sensing system, which has been used to detect the biomarkers in interstitial fluid. Microneedles-based sensors show a length of 1 mm, which leads to cause less pain than traditional injection needles [111]. Moreover, the tiny holes caused by microneedles can automatically heal within a few hours without bleeding and trauma. Many microneedles-based sensors have been developed to monitor health conditions [112,113,114]. For example, microneedle electrodes were prepared by combining 3D printing technology and Computerized Numerical Control micromachining technology using poly(methyl methacrylate) [112]. An aptamer has been used as a recognition component immobilized on the microneedle electrodes, which shows the molecular recognition ability toward the irinotecan, its major metabolite SN-38, and doxorubicin. The sensor can continuously monitor these drugs in real time in a wide detection range. Narayan and co-workers proposed a microneedle-sensing platform for fentanyl detection. The fentanyl in liquid samples was successfully detected using square-wave voltammetry with a detection range of 20~160 μM and an LOD value of 27.8 μM [114].

### 4.5. Contact Lenses Sensors

In recent years, contact lenses gradually developed from glass and plastic to soft hydrogels to obtain the functions of health detection [115]. The contact lens sensors are new detachable medical devices between “implantable” and “wearable”. Tears show compositional variations when suffering from ocular and systemic metabolic conditions. As such, contact lens sensors can be used to monitor health conditions by analyzing biomarkers in tears [116,117,118,119]. Microfluidic contact lenses were utilized for in situ sensing pH, glucose, protein, and nitrite ions in tears (Figure 3d) [116]. Four biosensors were located at the branch ends of the microfluidic device. The biomarkers were analyzed using a colorimetric method with a smartphone-MATLAB algorithm.

### 4.6. Mouthguard Sensors

Wearable biosensors can be integrated with mouthguards for monitoring dental health. There are plenty of metabolic molecules and bacteria in saliva closely associated with dental health [120]. For example, a uricase functionalized electrode was integrated with a mouthguard platform by a screen-printed technology, as show in Figure 3e [121]. The mouthguard biosensor exhibits high sensitivity and selectivity toward uric acid in human saliva, which can be used to monitor the health condition of hyperuricemia patients. Other biomarkers such as glucose, thiocyanate, H_2_S gas, and N-epsilon (carboxymethyl) lysine also can be detected using the same technology [122,123,124,125].

Various forms of wearable sensors have been designed to achieve real-time monitoring purposes such as patch type, fabric type, and tattoo type [126,127,128,129]. The application forms still need further investigation for practical use, which should consider stability, accuracy, and power supply. Meanwhile, the biocompatibility study on wearable sensors has been ignored for the last decade. Wearable sensors directly contact the human body, which may cause anaphylaxis or other uncomfortable feelings. Breathability, flexibility, stretchability, and biocompatibility are the most desirable features of wearable sensors to meet the requirements for real use.

## 5. Signal Processing

The sensing results cannot be directly used for health analysis, which requires further signal processing. Two aspects play a critically important role in signal processing: storage of the data collected from sensors and filtration of the collected data to improve the signal intensity. Optimization algorithms are often utilized for signal processing, which expands the application range and improves analysis accuracy. Convolutional neural network (CNN) is an efficient recognition method for signal processing, especially in the field of pattern classification. The CNN can use original images, avoiding complex image pre-processing. The CNN has been used for the automatic classification, acquisition, and analysis of ECG signals from wearable devices, which provides higher accuracy than other algorithms [130]. The CNN has been used to process the ultraviolet signals to quantify ultraviolet intensity for real-time ultraviolet monitoring [131]. Other signal-processing methods also have been utilized to analyze and process sensing signals [132,133]. The standard curve method is a typical processing procedure with a standard addition method [132].

Various signal processing methods are utilized to achieve important health information. In recent years, machine learning was used as a new signal processing method to achieve disease prediction functions [134,135]. The individual difference increases the complexity of signal processing and may affect disease analysis. Therefore, it is necessary to conduct a large number of tests in advance to improve accuracy for practical applications.

## 6. Wearable Biosensors

There are many kinds of biosensors based on human health monitoring, and their goals are different. Among them, the two main kinds are the physical sensor, which can detect temperature, pressure, strain and so on and the chemical sensor, which can detect the concentration of the target analyte. Here, the physiological pressure sensor of the physical sensor and the sweat sensor of the chemical sensor are mainly introduced. In addition, based on the wearable demand, the self-powered sensor without an external power supply is also an important kind of wearable sensor, which is also proposed in this section.

### 6.1. Physiological Pressure Sensors

Wearable physiological pressure sensors have gained tremendous interest in recent years due to their wide applications, which include but are not limited to detecting human emotion, diagnosing diseases, and monitoring health status. For physiological pressures, human intraocular and intracranial pressures are below 10 kPa, while others are often in the range of 10 to 100 kPa, including blood pressure, radial artery wave, and heart rate [58]. This fact means that pressure sensors should be elaborated to meet the requirements for measuring a specific type of pressure. So far, pressure sensors can be classified into piezoresistance, piezocapacitance, piezoelectricity, and triboelectricity. In this section, we will give a brief introduction to each mechanism and recent progress in physiological pressure sensors.

Piezoresistive pressure sensors own a simple structure so that they are ready to fabricate in labs and on a large scale, although extra power consumption is needed. For piezoresistive pressure sensors, the resistance (*R*) is mainly related to the materials’ resistivity (ρ), materials’ length (*L*), and the contact area (*A*):

(1) $$R=\frac{\rho L}{A}$$

**Figure 4:**
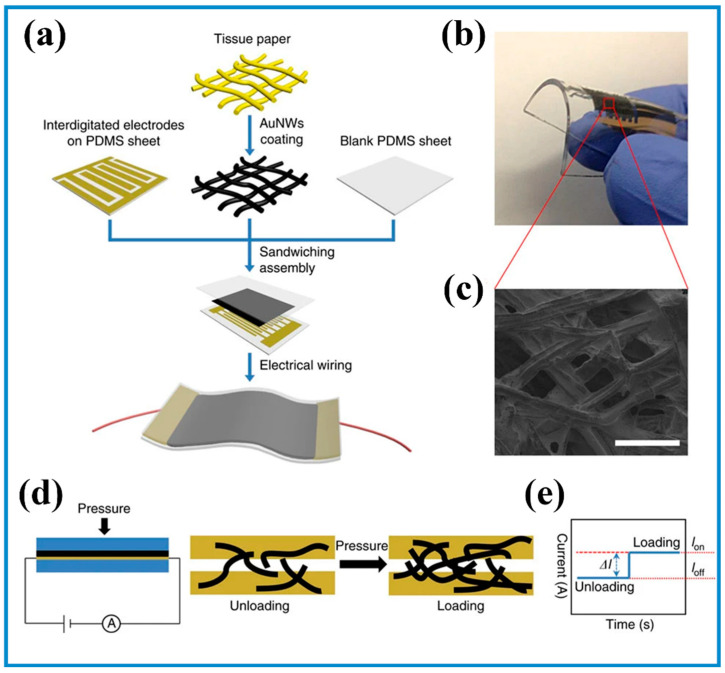
(**a**) Schematic illustration of the fabrication of a flexible sensor. Images showing (**b**) the bendability of the sensor, (**c**) the morphology of Au NWs-coated tissue fibers (scale bar = 100 μm). (**d**) Schematic illustration of the sensing mechanism. (**e**) Current changes in responses to loading and unloading. Reproduced with permission [140]. Copyright, 2014, Nature Publishing Group, a division of Macmillan Publishers Limited.

Typically, conductive fillers (e.g., carbon materials [136], metallic nanomaterials [137,138,139,140], and conductive polymers [141,142]) were added to elastomers (e.g., PDMS) to construct piezoresistive sensors. When pressure is applied to sensors, *R* is reduced as a result of a decrease in *L* and/or an increase in *A*. For instance, a MXene-sponge was fabricated by a simple dip-coating strategy [72]. An applied pressure resulted in more dense contact with each other, increasing the conductivity of the sensor. When the stress was released, the sponge returned to its initial state. In another case, Gong and colleagues reported a flexible piezoresistive pressure sensor by sandwiching ultra-thin Au nanowires (NWs)-impregnated tissue paper between a PDMS layer and a PDMS layer with patterned electrode arrays (Figure 4) [140]. External pressure forced Au NWs to contact the electrode arrays due to the compressive deformation of the paper. Meanwhile, the *L* of the sensor was reduced under applied pressure, leading to a decrease in *R* (Equation (1)). Therefore, the current was increased, showing a sensitivity of 1.14 kPa^−1^ in the range of 0–5 kPa at a voltage of 1.5 V. The gauge factor (GF) that was defined as the ratio of relative change in *R* to the strain was 7.38 when the strain was below 14%. Following this work, the same group sandwiched “soft” Au NWs and “rigid” Ag NWs between two PDMS layers by a consequential solution-processable approach. Compared to their early report [140], the sensor showed higher transparency (~60% of transparency at a visible wavelength of 550 nm), lower working voltage (0.1 V), and larger GF (~240) [137]. To increase the sensitivity of pressure sensors, piezoresistive pressure sensors were designed to own specific microstructures, which have been well reviewed by Bao and coworkers [143]. 

In addition, conductive hydrogels that feature three-dimensional networks are good candidates for the preparation of pressure sensors. The simplest way is to incorporate inorganic ions into hydrogels to increase conductivity [144,145]. Ge and co-workers prepared a transparent poly(acrylamide)-poly(vinyl alcohol) (PAAm-PVA) hydrogel with potassium chloride via a typical sol–gel method [144]. Under external pressure, the resistance decreased, and thus, the current increased. The sensitivity of the sensor is 0.05 kPa^−1^ below 3.27 kPa. Currently, hydrogel-based pressure sensors are usually able to detect many other deformations, such as twisting, stretching, bending, and others. In one case, Feng and co-workers reported a flexible hydrogel-based sensor that was composed of poly(N-isopropylacrylamide-co-AAm) (pNIPAm-co-AAm), PVA-graphene oxide (PVA-GO), and polyacrylic acid (PAAc)-Fe^3+^ ions [145]. The hydrogel has triple synergistic interactions in the gel matrix, showing a high compress strength of 1.1 MPa. The Fe^3+^ ions and rGO allowed the hydrogel an excellent conductivity (~170 Ω/mm). Under external pressure, the thickness of the hydrogel sensor changed, resulting in a rapid and reversible resistance change. In addition to sensing pressure, the hydrogel-based sensor was able to detect human skin temperature due to the temperature responsivity of pNIPAm. As a result of the collapse of pNIPAm at >32 °C, the sensor showed an enhanced resistance as a function of increasing temperature in the range of 30–55 °C. Taken together, the sensor showed pressure-temperature dual sensing capability [145]. In addition, incorporating conductive polymers and carbon nanomaterials are proven to be an effective approach to prepare hydrogel-based piezoresistive pressure sensors [146]. For applications in the real world, the evaporation of water in the hydrogel matrix should be effectively suppressed to maintain gels’ elasticity and thus sensing capacity. This concern can be solved by adding organic solvent or salts as well as elastomer encapsulation [147].

The capacitive sensors have also been used to monitor physiological pressure, which featured a dielectric layer sandwiched between two conducting electrode plates. Theoretically, the change in capacitance can be defined as

(2) $$C=\left(\varepsilon_{0}\times \varepsilon_{d}\right)\times A/d,$$

where *ε*_0_ and *ε_d_* are the permittivities of the vacuum and the dielectric layer, respectively, *A* is the contact area, and *d* is the distance between two conducting electrode plates. Therefore, changing *ε_d_*, *A*, and/or *d* can lead to a varied value of *C*. Soft elastomers [66,148,149,150,151,152], hydrogels [93,153], and inorganic films [154] have been used as dielectric layers in capacitive sensors. To increase the sensitivity, the morphologies of dielectric layers [70,148,149] or conducting electrodes [155,156] were designed into specific nano-/microstructures, such as pores [70,148], pyramids [9,157], pillars [158], and cones [149].

**Figure 5:**
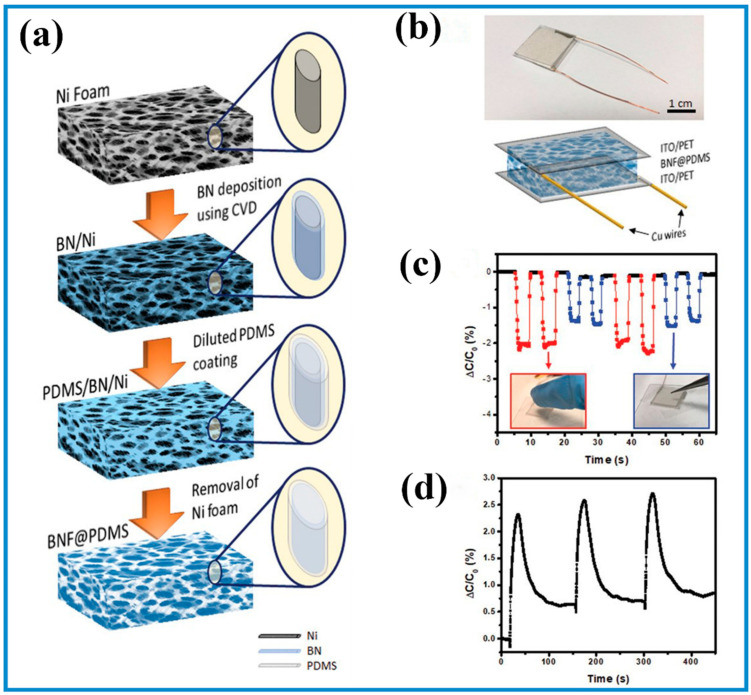
(**a**) Schematic of the fabrication process of BNF@PDMS. (**b**) Photograph (top) and schematic (bottom) of BN/PDMS-based capacitive sensor. Plots of ΔC/C0 as a function of applied (**c**) noncontact touch by hovering a finger (red) and a tweezer (blue) at close proximity to the device, and (**d**) environmental change by gently blowing through the device. The insets in (**b**) show the photographs of a hovering finger (boxed in red) and tweezer (boxed in blue) on top of the device [70]. Copyright, 2020 WILEY-VCH Verlag GmbH & Co. KGaA, Weinheim.

Changing *d* as a result of pressure can lead to a significantly varied value of *C*, while the synergetic effect due to changes in *ε_d_* and A should be considered. For instance, Tay et al. prepared a highly porous hexagonal boron nitride (BN)/PDMS foam that is lightweight (15 mg/cm^3^), and the *ε_d_* of the foam is close to that of air (Figure 5) [70]. Upon loading stress, the BN/PDMS foam was compressed, leading to a decrease in *d*, and thus, *C* is increased (Equation (2)). When releasing the load, *C* returned to its initial value because the BN/PDMS-based sensor returned to its initial state. Due to the synergistic effects between BN and PDMS, the sensor showed high compressibility (up to 95% strain), remarkably high sensitivity (0.854 kPa^−1^) at a pressure range of <0.5 kPa, and a lower LOD (<1 Pa). Another interesting example reported by Zhang and co-workers showed a wearable capacitive electronic skin composed of a pNIPAm-based microgel monolayer sandwiched between two silver soft electrodes [93]. The diameter and chemical composition of pNIPAm-based microgels affected the capacitance and thus the sensitivity of the sensors under external stress. Such a device can wirelessly sense pressure as low as 2 Pa, featuring a sensitivity of 10.1 kPa^−1^. Consequently, it was capable of monitoring pulse beat, apex beat, swallowing, etc. Differently, Xiong et al. demonstrated an ultra-highly sensitive capacitive sensor integrated by an ultra-thin dielectric layer and two micro-arrayed PDMS-Au electrodes. Under external pressure, the changes of *A* and *d* resulted in the superior properties of the sensor, showing a sensitivity of 30.2 kPa^−1^ and the LOD (0.7 Pa) in the pressure regime of <130 Pa [156].

The physiological pressure sensor has found wide applications in monitoring human activities and health conditions [159,160,161]. The sensitivity and detection pressure range are two important performances for practical applications. Sensitivity shows the sensor’s ability to identify external pressure signals, while the pressure range stands for the sensor’s application field. However, the sensitivity and detection pressure range exhibit a trade-off trend for most physiological pressure sensors. As such, the sensor with high sensitivity and a wide linear range has become a hot topic of research. In general, microstructures with a small form factor, such as sharp protrusions and micro-fluctuations, can generate a high sensitivity at low pressure, while structures with a large form factor lead to wider sensing ranges. The combination of the two concepts is capable of obtaining high sensitivity in a wide linear range.

### 6.2. Wearable Sweat Sensors

The body’s biochemical state is an important indicator in health monitoring. Blood tests are standard methods to determine the various biochemical indicators in the human body. However, blood sampling is an invasive and pain-produced procedure, which may lead to infection risk. Blood tests are thus not suitable for healthcare monitoring, which usually require daily or even time sampling. Sweat, one of the biological fluids secreted by eccrine glands through an osmotic pressure-driven manner, can be non-invasively collected on the skin’s surface [42]. A series of health indicators, e.g., pH, glucose, K^+^, Na^+^, Ca^2+^, and heavy metal ions, can be identified in sweat. Therefore, considerable efforts have been devoted to developing skin contact sensing devices, although the secretion of sweat needs to be activated.

#### 6.2.1. Sweat Extraction Strategies

Sweat collection is one important factor that affects wearable sweat analysis. In general, perspiration requires the assistance of exercise, heat, drug, and electric stimulation. The traditional sweat collection methods restrict the practical use of wearable sweat biosensors. Therefore, much effort has been devoted to exploring more efficient and convenient collection methods. Paper-based microfluidic channels have been demonstrated as an extremely effective method for long-term sweat collection and analysis. The porous structure of paper enables it to quickly absorb and transport sweat to specific locations through capillary pressure. Paper-based microfluidics can be integrated with wearable biosensors to achieve sweat analysis at rest [162,163]. For example, paper-based microfluidics were developed to deliver sweat fluid to the analytic area via capillary forces [162]. A hydrogel sheet was placed in a sampling well to extract sweat to microfluidic channels by interfacing it with the skin. The microfluidic device was integrated with a lactate-sensing strip to realize continuous lactate analysis. Textiles show excellent flexible and breathable properties, which enable it as a good candidate for sweat collection [164,165]. A textile was laser-engraved into tree-like bifurcating channels for sweat collection [164]. A fractal structure of the textile channels was designed to minimize sweat flow resistance. The textile-based microfluidic device shows a short induction time (<1 m) and a maximum flux of up to 4.0 μL cm^−2^ min^−1^ during sweat collection.

#### 6.2.2. Common Sweat-Based Wearable Sensor

- pH and Ion Detection

**Figure 6:**
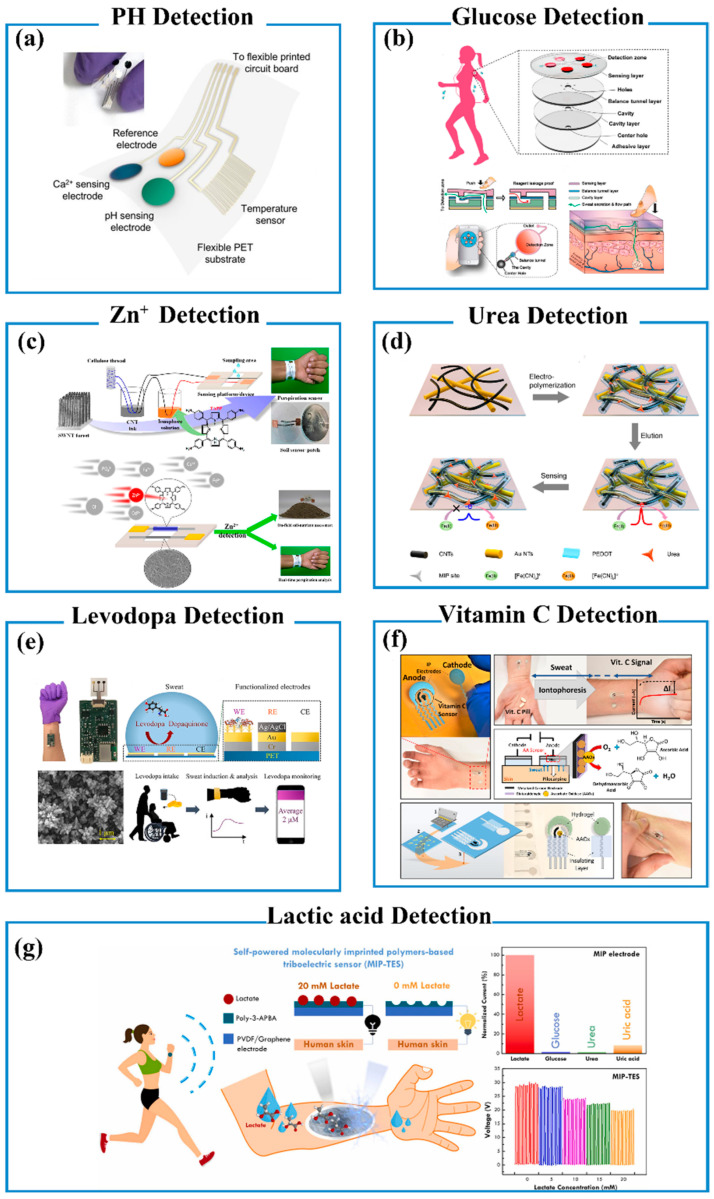
(**a**) A schematic diagram of a flexible sensor array consisting of Ca^2+^, pH, and temperature sensors patterned on a flexible PET substrate. Reproduced with permission [166]. Copyright 2016, American Chemical Society. (**b**) Schematic illustration of the sensor composed of four layers, including the five detection chambers containing the pre-embedded colorimetric reagents in the top layer. Reproduced with permission [170]. Copyright 2019, American Chemical Society. (**c**) Schematic description of the fabrication process of the wearable sensing device. Reproduced with permission [173]. Copyright 2019, American Chemical Society. (**d**) A schematic diagram of a flexible sensor to detect urea. Reproduced with permission [174]. Copyright 2018, American Chemical Society. (**e**) Schematic of the sweatband and levodopa sensing mechanism. Reproduced with permission [175]. Copyright 2019, American Chemical Society. (**f**) VC determination using wearable sensors in stimulated sweat. Reproduced with permission [177]. Copyright 2020, American Chemical Society. (**g**) Self-powered molecularly imprinted polymers-based triboelectric sensor (MIP-TES) for non-invasive lactate monitoring in human sweat. Reproduced with permission [179]. Copyright 2022, Elsevier Ltd.

The changes in pH are closely related to varied skin diseases such as dermatitis, ichthyosis, and fungal infections, while the concentration of alkali or alkaline–earth metal ions can reflect electrolyte homeostasis. Therefore, the pH and the concentration of ions in sweat are important analytes of wearable biosensors. Nyein and co-workers reported a wearable sensing system that was able to real-time monitor sweat pH, Ca^2+^, and skin temperature [166]. As shown in Figure 6a, the electrochemical platform was fabricated by logically imprinting a Ca^2+^ sensor, a pH sensor, and a skin temperature sensor on a flexible printed circuit board. The system allowed the accurate determination of Ca^2+^ and pH in body fluids including sweat, urine, and tears. The real-time Ca^2+^ and pH simultaneous detection not only minimized cross-contamination from delayed samples but also avoided pH correction in clinical diagnosis, e.g., hypercalcemia and hypocalcemia tests. In another case, Nakata et al. developed a mechanically flexible sweat pH sensor using an InGaZnO-based ISFET (ion-sensitive field-effect transistor) [167]. The pH value of sweat and skin temperature could be recorded in real time by attaching the device to the neck of testers during exercise. Cordero et al. showed a fully integrated sweat-sensing device, consisting of state-of-the-art ISFETs, ion-sensing membranes (ISMs), miniaturized quasi-reference electrode (QRE), and low-volume passive microfluidics [168]. The embedded QRE elevated the sensing platform with excellent linear sensitivity. During the real-time measurements, the platform was also able to perform stable and repeatable readings, which could fully track the physiological variation in ion concentrations in sweat.

- Glucose Detection

The uncontrollable blood glucose level caused by diabetes generates the requirements for daily blood measurement. Recently, considerable attention has been paid to monitor sweat glucose using wearable sensors due to their non-invasive and painless advantages. For example, Zhao and co-workers demonstrated a proof-of-concept wearable glucose biosensor based on a highly stretchable gold fiber for monitoring glucose in sweat [169]. The wearable sensor is a three-electrode electrochemical platform, where a gold fiber with Prussian blue and glucose oxidase was used as a working electrode, an Ag/AgCl electrode was used as a reference electrode, and a bare gold fiber was used as a counter electrode. The glucose in the sweat sample was oxidized with the catalysis of glucose oxidase on the working electrode to produce H_2_O_2_. Cyclic voltammetry was utilized to measure the concentration of H_2_O_2_ generated from the oxidization of glucose in human sweat. In addition, the intrinsic stretchability of the gold fibers was further strengthened by forming a helix structure, resulting in a robust electrochemical performance with strains up to 200%. Xiao and co-workers reported a microfluidic chip-based wearable colorimetric sensor for detecting sweat glucose, as shown in Figure 6b [170]. The sensor exhibited benign practical applications in detecting glucose in sweat samples from a group of volunteers engaged in both fasting and postprandial trials. The sensor contains glucose oxidase, peroxidase, and o-dianisidine, where the o-dianisidine was used as a colorimetric reagent. During the detection, glucose in sweat was oxidized with the catalysis of glucose oxidase to produce H_2_O_2_. The produced H_2_O_2_ further oxidized the colorless o-dianisidine into red-colored oxidized o-dianisidine with the catalysis of HRP. The obtained sensors for detecting glucose in sweat showed a good linear range from 0.1 to 0.5 mM with an LOD of 0.03 mM. He and co-workers fabricated an intelligent Janus textile band, which was integrated with a self-pumping sweat collection, comfortable epidemic microclimate, and sensitive electrochemical biosensing [171]. The sweat pumping was driven by Janus wettability, which is realized by electrospinning a hydrophobic polyurethane (PU) nanofiber array onto a super-hydrophilic gauze. The sweat could thus be unidirectionally and thoroughly transported from the skin (hydrophobic side) to the embedded electrode surface (hydrophilic side). The electrode could sensitively detect metabolites in sweat such as glucose, lactate, K^+^, and Na^+^ in real time. Moreover, the wearable device leads to physiological comfort due to the effective drain of epidermal sweat. Glucose oxidase can catalyze the oxidation reaction of glucose, yielding H_2_O_2_ molecules. Thus, sweat glucose levels can be measured by H_2_O_2_ signals. For example, Li and co-workers fabricated a wearable sweat-capture device using patterned graphene arrays with controlled superwettability and electrical conductivity, which could simultaneously capture sweat droplets and electrochemically measure glucose [172]. Glucose detection is also involved with glucose oxidase-dependent enzymatic reaction.

- Zn^2+^ Detection

Zn, an important element involved in various enzyme-relevant biochemical processes, plays a wide variety of physiological roles such as maintaining normal metabolism and immune function. Zn concentration in biological fluids offers a reflection of broad physiological states, e.g., muscle damage. Considerable challenges have been encountered in the non-invasive detection of Zn^2+^ due to microliter sampling volumes and sub-ppm concentrations (0.39–1.56 ppm). Thus, there are long-term pursuits of wearable Zn^2+^ sensors with high sensitivity, specificity, a wide dynamic range, high signal reliability, and real-time detection from low-sampling volumes. Subramaniam and co-workers developed a miniaturized, coaxial, cable-type electrochemical sensor, consisting of a carbon nanotube (CNT)-immobilized cellulose yarn, as shown in Figure 6c [173]. The sensor comprises two cables of CNT thread. One cable was coated with a polymeric ion receptor, tetrakis(p-aminophenyl) porphyrin (TAPP), which acted as a working electrode, while the other cable without modification was used as a reference electrode. The TAPP-receptor membrane interacted with Zn^2+^ through Faradaic charge-transfer interactions, which demonstrates the ability to selectively capture Zn^2+^ from solutions. The Zn^2+^ concentration could be measured through electrochemical techniques, such as cyclic voltammetry, differential pulse voltammetry, and chronoamperometry. The wearable sensor had a rapid response (~60 s) toward Zn^2+^ with ultra-high sensitivity in the range from 0.1 to 500 ppm. It showed superior anti-interference (selectivity coefficient 10^−3^–10^−5^) from various cations (Na^+^, K^+^, Mg^2+^, Cd^2+^, Ca^2+^, Fe^2+^, and Cu^2+^) and anions (Cl^−^, NO^3−^, PO_4_^3−^, and CH_3_COO^−^). The practical performance also had been validated by the real-time detection of Zn^2+^ across a varied spectrum of samples ranging from human perspiration to agricultural soil.

- Urea Detection

Urea, an important nitrogen metabolite in body fluids, is one of the important indicators of renal function and heart metabolic disorders. High levels of urea in sweat often appear in patients with hepatic or renal failure, or uremia, indicating the importance of monitoring sweat urea. Liu and co-workers reported a flexible and conformal urea sensor based on a molecularly imprinted polymer (MIP) approach, as shown in Figure 6d [174]. Firstly, the urea-targeted poly(3,4-ethylenedioxythiophene) (urea-PEDOT) MIP was deposited on a hybrid nanostructure of CNT and gold nanotube (Au NTs) through electropolymerization. Then, the urea template molecules in the MIP were removed to obtain the molecularly imprinted recognition sites, which showed the ability of specific urea recognition. The urea molecules in the sweat sample could rebind with molecularly imprinted recognition sites during the urea detection. The MIP sensor exhibited a good linear response toward physiological levels of urea without interferences from common coexisting species. Furthermore, this flexible sensor possessed excellent mechanical tolerance and exhibited unaffected electrochemical performance under bending deformation. The sensors could distinctly respond to the urea level variations in volunteers’ sweat after aerobic exercise, which validates their practical values.

- Levodopa Detection

Levodopa, the precursor of dopamine, has been treated as the standard medication clinically prescribed to people with Parkinson’s disease. The levodopa dosage monitoring and controlling is remarkably critical due to the dosage-dependent onset of undesired fluctuations in the patient’s physical and emotional conditions, e.g., speech movement and mood. Additionally, levodopa undergoes a xenobiotic metabolism pathway and can excrete through sweat. Thus, recent attention has been paid to the development of wearable sensors for sensing sweat levodopa. Tai and co-workers fabricated a wearable sweatband on a nanodendritic electrochemical platform for quantitatively monitoring levodopa, as shown in Figure 6e [175]. The sensing element of the sweat levodopa band is a standard three-electrode electrochemical platform, where the working electrode was functionalized with tyrosinase enzyme. The levodopa was oxidized with the catalysis of tyrosinase enzyme to dopaquinone, which generated the Faradaic current. The Faradaic current was measured to evaluate the concentration of levodopa in sweat samples. The robustness and stability of the electrochemical sensor were greatly enhanced by the seamless incorporation of dendritic growth, enzyme immobilization, and stabilizing film. The practical application of the sweatband for long-term and non-invasive monitoring levodopa was undertaken in human subjects after fava beans intake, showing the desired guidance for dosage optimization. Similarly, Javey and co-workers reported a wearable PDMS-based microfluidic device integrated with electrochemical electrodes for the continuous measurement of sweat composition such as pH, Cl^−^, and levodopa [176]. The device mainly consisted of sweat-sensing electrodes, a laminated hydrophilic filler, and a PDMS-based microfluidic layer three elements. The sensing electrode of levodopa was prepared by depositing the tyrosinase enzyme on Au nanodendrites. The levodopa was oxidized with the catalysis of tyrosinase enzyme to dopaquinone generating the Faradaic current, which was used to measure the concentration of levodopa in the sweat sample.

- Vitamin C (VC) Detection

VC is a very important physiological reductive molecule, which can easily be absorbed from normal fruits, e.g., oranges. VC is involved in abundant physiological activities, e.g., collagen production (related wound healing and skin care), iron absorption, cold treatment, immune system, and neurological disorders. Sempionatto and co-workers developed a wearable electrochemical biosensor that monitored the concentration and dynamics of VC in a non-invasive manner, as shown in Figure 6f [177]. Such wearable noninvasive sensors were fabricated by immobilizing ascorbate oxidase on flexible printable tattoo electrodes. The resultant wearable biosensor could reliably measure the temporal rise and fall of sweat VC concentrations in sweat samples with high sensitivity after taking VC pills or juices. Similarly, Javey and co-workers reported an Au nanodendrites-based three-electrode electrochemical platform, where the Au nanodendrite with conducting PEDOT doped with LiClO_4_ and L-ascorbate oxidase was used as a working electrode [12]. When VC presents in sweat samples, the VC will be oxidized by O_2_ with the catalysis of L-ascorbate oxidase, which generates the Faradaic current for measuring VC concentration. In another case, Kim and co-workers reported a wireless, skin-interfaced microfluidic biochemical device that contained immunoassays for sweat cortisol and fluorescent assays for glucose and VC, respectively [178]. The microfluidic structure was used to reduce the sweat evaporation rate and collect sweat in a rest state. The collected sweat was stored in a pair of reservoirs connected by microchannels with VC oxidase and glucose oxidase. The oxidation reactions of VC and glucose generated H_2_O_2_ that oxidized a fluorometric probe (OxiRed) to form resorufin, yielding a fluorescence signal.

- Lactic Acid Detection

Lactic acid is an important biomarker produced by the human body during anaerobic exercise causing muscle soreness and other conditions. The lactic acid level in sweat also can be monitored using wearable biosensors. Molecularly imprinted technology has been used to detect lactic acid in sweat to increase selectivity [179]. In one case, lactate molecules imprinted with poly(3-aminophenyl boronic acid) have been used as a selective electrode, which exhibited the selective lactate-sensing performance and stable signal changes with lactate concentration variations. Lactic acid also can be detected by traditional enzyme catalytic reactions [180]. Lactate oxidase was immobilized on the electrode surface, which can convert lactate to pyruvate, yielding hydrogen peroxide. Then hydrogen peroxide can be detected by electroanalytical methods using Prussian blue as a redox mediator. In addition to the aforementioned methods, lactic acid also can be detected by measuring the variation in sweat pH [181].

#### 6.2.3. Problems That Occur When Analyzing Sweat

Although wearable sweat sensors show tremendous advantages over blood-based diagnosis, there are still some critical challenges to overcome for practical use. Firstly, sweat collection technologies at rest need to be further explored for convenient use. In the current stage, sweat collection requires the assistance of exercise, drugs, or electro stimuli, restricting its real use. Secondly, the concentration of biomarkers in sweat is much lower than in vivo concentration. As such, it requires wearable sweat sensors with much higher sensitivity. More innovative materials should be designed and prepared to enhance the sensing performance. Last, sweat evaporation will lead to inaccurate results during biomarker analysis. The sweat excreted at different time intervals may mix, which also results in inaccurate results.

### 6.3. Wearable Self-Powered Biosensors

Flexible and wearable biosensors have enormous benefits for monitoring a health condition. Wearable biosensors require batteries to offer energy for sensing. The development of self-powered biosensors provides a possible approach to avoid the inconvenience caused by batteries. The wearable self-powered biosensors can harvest energy from the body or ambient atmosphere and convert it into electrical signals. There are three types of self-powered biosensors classified by operating mechanisms, such as piezoelectric nanogenerators (PENGs) [182,183,184], triboelectric nanogenerators (TENGs) [185,186,187], and pyroelectric nanogenerators (PyNGs) [83]. In this section, we mainly describe the working principles and applications of various wearable self-powered biosensors in the health-monitoring system [188].

#### 6.3.1. Piezoelectric Nanogenerators (PENGs)

**Figure 7:**
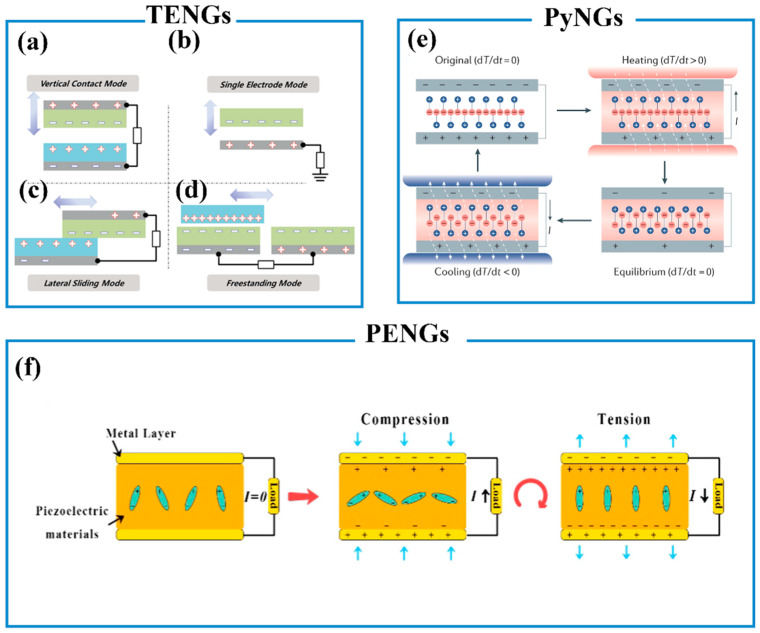
The four fundamental modes of TENGs: (**a**) Vertical contact mode, (**b**) Lateral sliding mode, (**c**) Single electrode mode, (**d**) Free-standing triboelectric layer mode. Reproduced with permission [197]. Copyright 2018, WILEY-VCH Verlag GmbH & Co. KGaA, Weinheim. (**e**) Working mechanism of PyNGs. Reproduced with permission [2]. Copyright 2020, Springer Nature Limited. (**f**) Working mechanisms of PENGs. Reproduced with permission [188]. Copyright 2020, Elsevier Ltd.

PENGs consist of a piezoelectric layer and two electrodes, which is an important energy-harvesting device based on the polarization of piezoelectric materials (Figure 7f) [188]. When an external force is applied, equal but opposite charges will be generated on the tensile side surface and compressive side surface due to the polarization of the piezoelectric layer. Therefore, an electron flow is generated from the bottom to the top electrode of the device [189]. The typical piezoelectric materials include polyvinylidene fluoride (PVDF) [190,191], lead titanate, [192] and gallium nitride [193]. Many strategies have been proposed to enhance the performance of PENGs such as promoting mechanical drawing, adding nanofillers [191], and combining with transistors [192].

**Figure 8:**
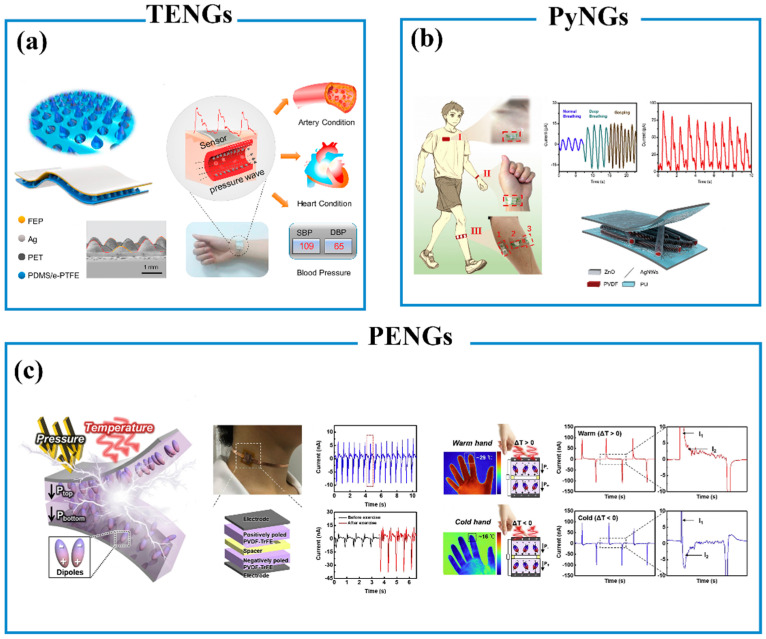
(**a**) Schematic diagram of a TENG-based biosensor. Reproduced with permission [202]. Copyright 2020, Elsevier Ltd. (**b**) Schematic diagram of a PENG-based biosensor. Reproduced with permission [195]. Copyright 2020, Elsevier Ltd. (**c**) Schematic diagram of TENG/PyNG-based biosensor. Reproduced with permission [203]. Copyright 2020, Elsevier Ltd.

PENG-based biosensors have a similar structure to TENG-based biosensors, which achieved self-powering performance based on the polarization of piezoelectric material. PENG-based biosensors exhibited potential applications in human physiological health monitoring [184]. PVDF is the most well-known semi-crystalline piezoelectric material with strong piezoelectricity, which has been used to fabricate PENG-based biosensors [194]. For example, Deng and co-workers fabricated a PENG-based hierarchically interlocked PVDF/ZnO nanofibers biosensor by growing the ZnO nanorods on the surface of PVDF nanofibers (Figure 8b) [195]. The sensor was fabricated by sandwiching PVDF/ZnO nanofibers with two PU films embedded with Ag NWs. The PU/Ag NWs films and PVDF/ZnO nanofibers were used as electrodes and a piezoelectric layer, respectively. When an external force is applied to the sensor, equal but opposite charges will be generated on the two surfaces of the PVDF/ZnO nanofibers due to the polarization of PVDF/ZnO nanofibers. This process generates charge flow between the top and bottom electrodes. The sensor was used to monitor physiological signals such as walking, respiration, wrist pulse, and muscle behavior. Similarly, Chen and co-workers reported a flexible and wearable PENG-based 3D cellular sensor array, which contained a 3D cellular electrode with caged piezoelectric nanoparticles [196]. The piezoelectric functional layer was sandwiched by two copper electrodes. The sensor exhibited high-pressure sensitivity of 0.19 V kPa^−1^ and a fast response time of 16 ms, which has been used to monitor the human heartbeat and eyeball motion.

#### 6.3.2. Triboelectric Nanogenerators (TENGs)

TENGs were firstly reported by Wang and co-workers in 2012, which generated charges by two materials with different electronegativity contacting and separating based on the principle of triboelectrification and electrostatic induction [198]. When two materials with different electronegativity are in contact with each other, equal but opposite triboelectric charges are generated on these materials’ surfaces. If the two surfaces of materials are separated, a potential drop will be created, resulting in a flow of electrons between two electrodes [199]. According to the polarization change and electrode configuration, there are four fundamental working modes of TENGs: vertical contact mode, single electrode mode, lateral sliding mode, and freestanding mode [197]. The vertical contact mode has been shown in Figure 7a, in which two friction materials are placed face to face. When an external force was applied to the sensor, the direct contact of two materials resulted in equal but opposite charges and potential in the vertical direction [198]. The single electrode mode contains an electrode layer and a moving layer, which is the most simple mode (Figure 7b). The electrical field will be changed between the electrode and the moving layer due to the periodic contact and separation. This change results in a flow of electrons between the ground and the electrode to match the potential difference [200]. The lateral sliding mode has a similar structure to the vertical contact mode based on two different materials with metal coatings on the back sides (Figure 7c). Equal but opposite charges will be generated on the surface of the materials when two materials slide with each other. Then, when the top layer moves over the base layer, a flow of electrons occurs between the bottom and top electrodes until reaching equilibrium states [201]. The freestanding mode is composed of a moving layer and two electrodes. The charge will be generated due to the triboelectrification between the moving layer and two electrodes. An electron flow from one electrode to another occurs due to the potential generated to balance the asymmetric charge (Figure 7d) [83].

The TENG-based biosensor has been widely investigated for monitoring health conditions and disease prediction. It can convert the human body and internal organ motions into electrical signals based on the contact electrification and electrostatic induction between two different electron-affinity materials [204,205]. TENG-based biosensors have been used as biomedical and healthcare devices to detect physiological signals in the health-monitoring system. For example, Hu and co-workers reported a wearable self-powered pressure sensor for cardiovascular healthcare (Figure 8a) [202]. The sensor was fabricated by sandwiching conical PDMS-based hierarchical elastomer microstructures (HEM) between two ultra-thin films of fluorinated ethylene propylene (FEP)/Ag film and Ag/polyethylene terephthalate film, respectively. When an external force was applied to the sensor resulting in the deformation of the PDMS elastomer, the top film contacted the bottom film, resulting in the electrons flowing from the bottom electrode to the top electrode. Once the pressure is released, the PDMS elastomer rebounds to its original state, leading to reverse charge flow. This sensor achieves a sensitivity of 7.989 V kPa^−1^ in the pressure range of 0.1–60 kPa. In addition, the sensor without an external power source can not only monitor pulse, artery, and heart condition but also monitor the wide range of blood pressure. The sensing performance of TENG-based sensors depends on the interfacial interaction of two materials and charge generation on the contacting layers. As similar to many sensors mentioned in this section, TENG-based sensors that featured microstructures on the surface usually showed enhanced sensitivity, including pyramides, nanopillars, and microneedles [206,207,208,209]. To solve the complexity rising from traditional fabrication procedures, Ke and co-workers reported a simple CO_2_ laser to build an Al/PDMS triboelectric pressure sensor that showed the sensitivity of ~3.11 V kPa^−1^ due to the high pattern density and contact area caused by the unique two-height microneedles array [208]. Drawing inspiration from human skin haptic performance, Maharjan and co-workers designed a soft piezoelectric pressure sensor that owned interlocking microstructures. The sensor can sense a wide range of pressure from 0.2 to 500 kPa with a sensitivity of 0.77 V kPa^−1^ [209]. The triboelectric effect also can be coupled with enzymatic reactions to realize multiple sensing functions. For example, Liu and coworkers fabricated stretchable fiber-based TENG with stretchable conductive fibers and varnished wires [210]. The fibers were modified with glucose oxidase, lactate oxidase, and creatininase, which enable the TENG to precisely sense the motion states, glucose, creatinine, and lactate in sweat.

#### 6.3.3. Pyroelectric Nanogenerators (PyNGs)

PyNGs are energy-harvesting devices that can convert thermal energy into electrical energy based on the spin Seebeck effect (Figure 7e) [2]. The pyroelectric effect is related to the spontaneous polarization of pyroelectric materials. When temperature increases or decreases over time, the intensity of spontaneous polarization (Ps) will decrease or increase, respectively [189]. In addition, the short-circuit current and open-circuit voltage of pyroelectric materials can be evaluated using Equation (3) and Equation (4), respectively [211].

(3) $$I_{se}=\frac{Q}{∆T}=P∆T,$$

(4) $$V_{oc}=\frac{Q}{C}=\frac{P}{\varepsilon_{33}^{T}}\cdot h\cdot ∆T,$$

where *Q* is the pyroelectric charge, *p* is the pyroelectric coefficient, *A* is the surface area, and ∆*T* is the change of temperature. In addition, the stored electrical energy of pyroelectric materials can be evaluated using Equation (5) [211]:

(5) $$E=\frac{1}{2}\cdot \frac{P^{2}}{\varepsilon_{33}^{T}}\cdot A\cdot h\cdot ∆T^{2}.$$

Meanwhile, the electrical energy depends on the pyroelectric coefficient, which can be evaluated using Equation (6) [212]:

(6) $$P=\frac{dP_{s}}{dT}.$$

Multifunctional biosensors have great significance in the field of wearable health-monitoring devices. Multiple self-powered biosensors can be integrated into one device such as TENG/PENG-based biosensors [213], TENG/PyNG-based biosensors [203], and PENG/PyNG-based biosensors [189]. The TENG/PyNG-based biosensor can convert thermal energy and mechanical energy into electrical signals based on the pyroelectric effect and triboelectric effect without any interference. It is one of the important self-powered multifunctional biosensors for monitoring health conditions in disease prediction systems. For example, Ko and co-workers reported a self-powered multimodal pressure–temperature sensor based on P(VDF-co-TrFE) (Figure 8c). The sensor shows high-pressure sensitivity of 40 nA kPa^−1^ and 1.4 V kPa^−1^ in the pressure range of 98 Pa–98 kPa and temperature sensitivity of 0.38 nA °C^−1^ and 0.27 nA °C^−1^ in cooling and heating states, respectively. In addition, this sensor can be used to monitor pulse pressure as well as multimodal finger touch [203].

#### 6.3.4. Biofuel Cell

In recent years, biofuel cells have attracted much attention due to their capability of converting biomass energy into electricity by bioelectrochemical reactions [214,215]. In general, biofuel cells are comprised of an anode, a cathode, and an electrolyte. The biofuels are oxidized on the anode catalyzed by enzymes or microbial to generate electrons. Electrons are transmitted to the cathode through external circuits with the consuming biofuels [216]. The intensity of electric signals is dependent on the concentration of biofuels. Biofuel cells have been used to sense various metabolites in sweat including glucose, growth factor, lactate, ethanol, etc. [217,218,219,220]. Since the deactivation or leaching of enzymes occurs with time, signal stability is a significant challenge for biofuel cells during long-term use. In one case, laccase was encapsulated in a porous organometallic framework material to enhance enzyme stability [221]. The composite was trapped in a bacterial cellulose/carbon nanotube framework to prepare a highly flexible anode of biofuel cells. The biofuel cell is capable of self-powered sensing phenolic compounds. In another case, a wearable microbial fuel cell was fabricated using bacillus subtilis as dormant biocatalysts, which yielded a maximum power density of 16.6 μW/cm^2^ through bacterial activity by consuming lactic acid in sweat [222]. The microbial fuel cell shows no significant decrease in electrical output after 3 weeks of storage.

To increase the capability of practical use, wireless communication technology was utilized in self-powered sensing systems [223,224]. For example, a self-powered wearable sensing system can be used for on-demand, continuous, and real-time pulse monitoring, which includes a TENGs power supply unit, pressure sensor, and multifunctional circuit. It shows the capability of power management, energy storage, data collection, visualization, and wireless data transmission. The device exhibits an output power of 816.6 MWm^−2^, a sensitivity of 6.03 kPa^−1^, a low detection limit of 9 Pa, and a response time of 80 ms [223]. In another example, a self-powered wearable system for telemedicine applications has been fabricated including three units: a self-powered unit consisting of a diode and a capacitor, sensor data acquisition, and remote monitoring. The sensor acquires health parameter data from different parts of the body and sends it to a microcontroller. The microcontroller is coupled with a Wi-Fi module for data communication with a cloud-based website displaying real-time health parameters through the website [224].

Self-powered sensing systems can run without an external power supply recognized as a new wearable sensing manner [225,226,227]. A series of electricity generation mechanisms have been investigated to improve the portability of the sensing system such as triboelectricity [201], piezoelectricity [194], and hydrovoltaic effect [38]. The trade-off between sensitivity and output power is a main issue for self-powered sensing systems. Some new methods have been developed to improve both sensitivity and power output. In addition, long-term durability is another large issue to overcome for self-powered sensing systems.

## 7. Summary and Future Prospects

Flexible and wearable biosensors find various important applications in the field of medical treatment and health. It can enable continuous health monitoring and convenient diagnosis in real time. This is particularly important for monitoring various health risks in people’s daily life. In this review, recent advances in flexible and wearable biosensors are discussed, focusing on principles, techniques, and applications. Various microfabrication techniques are introduced in this paper, which benefits to improve the performance of biosensors. However, there are many challenges and limitations of biosensors to overcome for further application and commercialization. For example, the limited resolution of micro-nanofabrication technologies leads to large difficulties with preparing multifunctional integrated biosensors. In addition, various deposition technologies are utilized to fabricate sensing electrodes, which reduce the flexibility of devices and the stability of output signals. The mechanical mismatch between an electronic device and the biological tissue is another large issue when it comes to obtaining stable signals. In general, the sensing electrodes show much larger elastic modulus than biological tissues, which results in inferior contact interfaces generating signal losses. As such, more efforts are encouraged to devote in this field to overcome the current challenges and inspire more creative applications.

## Data Availability

Not applicable.
